# Operation spinal cord regeneration: Patterning information residing in extracellular matrix glycosaminoglycans

**DOI:** 10.1002/brb3.1531

**Published:** 2020-01-16

**Authors:** Alexander Lu, Alaina Baker‐Nigh, Peng Sun

**Affiliations:** ^1^ Department of Biology Saint Louis University St. Louis Missouri; ^2^ Program in Neuroscience Saint Louis University St. Louis Missouri; ^3^ Department of Neurosurgery Affiliated Hospital of Qingdao University Qingdao China

**Keywords:** axolotls, chondroitin sulfate, glycosaminoglycans, heparan sulfate, spinal cord regeneration

## Abstract

**Introduction:**

Spinal cord injuries are devastating, with many complications beyond paralysis and loss of sensory function. Although spinal cord regeneration can revolutionize treatment for spinal cord injuries, the goal has not yet been achieved. The regenerative mechanism of axolotls demonstrates that the regeneration is a repeat of developmental process that all animals have all the genes, but axolotls have both the genes and the patterning information to do it at the adult stage.

**Methods:**

A narrative review was conducted. Relevant studies were collected via an English‐language PubMed database search and those known to the authors.

**Results:**

Research during the past 30 years reveals that growth factors, along with spinal cord extracellular matrix, especially glycosaminoglycans, regulates axonal regrowth. Degrading chondroitin sulfate glycosaminoglycans by injecting the bacterial enzyme chondroitinase improves axonal sprouting and functional recovery after spinal cord injury in both rodents and rhesus monkeys. Furthermore, the brain is one of the first organs to develop during the embryonic period, and heparan sulfate glycosaminoglycans are key molecules required for brain development.

**Conclusions:**

Patterning information residing in glycosaminoglycans might be key elements in restricting spinal cord regeneration. A recommended solution is not to edit the human genome, considering the conserved signaling pathways between animals, but to take advantage of the regenerative mechanism of axolotls and the current knowledge about the pattern‐forming glycosaminoglycans for successful spinal cord regeneration and clinical applications.

## Graphical abstract

Spinal cord injuries are devastating, with many complications beyond paralysis and loss of sensory function. A recommended solution is to take advantages of the regenerative mechanism of axolotls and the current knowledge about the pattern‐forming glycosaminoglycans for successful spinal cord regeneration and clinical applications.

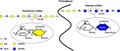

## 1 INTRODUCTION

What are glycosaminoglycans? How do they relate to spinal cord regeneration? The major glycosaminoglycans include heparan sulfate, chondroitin sulfate, and keratan sulfate, which are primed on the core proteins of proteoglycans. Glycosaminoglycans interact with hundreds of extracellular growth factors, chemokine, cytokines, proteases, and protease inhibitors and are essential for animal development (Inatani, Irie, Plump, Tessier‐Lavigne, & Yamaguchi, 2003; Lin, 2^2004^; Poulain & Yost, 2015; Swarup, Hsiao, et al., 2013; Zhang, 2010). Such interactions are glycosaminoglycan‐dependent because removing most of the core proteins of the proteoglycans does not affect the development, whereas knocking out glycosaminoglycan modification enzymes in various animal models are detrimental to development (Hayes & Melrose, 2018; Townley & Bulow, 2018). Furthermore, specific sulfation patterns in heparan sulfate and chondroitin sulfate are not only responsible for specific biological activities but also for axonal growth or inhibition (Griffith et al., 2017; Sakamoto et al., 2019; Shukla, Liu, & Blaiklock, 1999; Zhang et al., 2001), which suggest that the patterning information residing in glycosaminoglycans might be crucial for spinal cord regeneration.

Spinal cord injuries (SCIs) are devastating to a person's life. In 2018, there will be an estimated 17,700 new SCI cases in the United States, with the main causes of SCIs being car accidents, violence, sports injuries, and falls, especially among elderly patients (National Spinal Cord Injury Statistical Center, 2018). Alarmingly, the number of SCI cases due to falls in elderly patients increased from 28% in 1997–2000 to 66% 2010–2012 (Jain et al., 2015). This trend is expected to increase with an aging population (DeVivo, 2012).

Currently, there is no effective treatment for full recovery from SCIs, with most of the care being palliative (Tran, Warren, & Silver, 2018). Although there are advances in therapy such as stem cells, understanding the biological mechanisms behind SCIs and regeneration is needed (Bryant & Gardiner, 2018). It has been realized that in both the central nervous system (CNS) and peripheral nervous system, axonal regeneration is more dependent on the extracellular matrix (Swarup, Mencio, Hlady, & Kuberan, 2013). Early research on using “bridges” made from peripheral nervous system components helped researchers understand that the CNS axons can regenerate if placed in a permissible environment (David & Aguayo, 1981), demonstrating the importance of microenvironment.

Perineuronal nets (PNNs) are lattice‐like extracellular matrix structures mainly composed of chondroitin sulfate proteoglycans (Fawcett, Oohashi, & Pizzorusso, 2019; Jones, Margolis, & Tuszynski, 2003). Indeed, the research during the past 30 years reveals that specific glycosaminoglycan structures regulate axonal guidance and regrowth (Emerling & Lander, 1996; Swarup, Hsiao, et al., 2013). Moreover, degrading chondroitin sulfate glycosaminoglycans by injecting the bacterial enzyme chondroitinase improves axonal sprouting and functional recovery after spinal cord injury in both rodents and rhesus monkeys (Carter et al., 2008; Rosenzweig et al., 2019). Furthermore, N‐sulfated heparan sulfate mimetics promote myelination. In contrast, O‐sulfated heparan sulfate mimetics do not affect myelination but promote neurite outgrowth (McCanney et al., 2019). Thus, both chondroitin sulfate and heparan sulfate glycosaminoglycans play important roles in axonal regeneration.

Current knowledge of SCIs is mainly derived from animal models, especially rodents and nonhuman primates. Rodents are commonly used for its availability and number of interventions possible, but their SCI models are different from actual SCIs in humans (Nardone et al., 2017). Ma et al. (2016) utilized a spinal contusion model for rhesus monkeys. The advantage of such a model is the physiological and genetic similarities between monkeys and humans, and the disadvantages are the high cost and the replicability. However, a preclinical study has been conducted successfully by using rhesus monkeys as a model system (Rosenzweig et al., 2019; Steward & Willenberg, 2017).

Understanding spinal cord regeneration in nonmammals is important to identify what is needed for successful regeneration and possible application of their mechanisms for future clinical therapies. The potential nonmammal model of SCIs is axolotl due to their ability to regenerate their spinal cord with full functionality (Tazaki, Tanaka, & Fei, 2017). Compared with mammals, an SCI to axolotls result in total repair of the spinal cord (Rost et al., 2016). Based on regeneration studies, it has been revealed that both pattern‐following and pattern‐forming cells are required (Bryant & Gardiner, 2018). Great efforts have been made toward understanding the pattern‐following cells, especially stem cells, but little is known about the nature of pattern‐forming cells and the patterning molecules. Using axolotls as a model, it is found that patterning mechanisms in development and regeneration are the same (Muneoka & Bryant, 1982) and the number of genes in axolotls is similar to what in humans (Nowoshilow et al., 2018), indicating the regeneration capability of axolotls rely on their capacity to repeat developmental process using the same set of genes. Therefore, glycosaminoglycans that contain both genetic and environmental information such as the status of nutrients, vitamins, minerals, and oxygen supplies in a time‐ and space‐dependent manner might be the sought‐after elements that limit spinal cord regeneration in mammals.

This review will focus on our current understanding of spinal cord injury mechanisms and discuss how to counter the negative effects of those mechanisms and current limitations. A recommended solution is to take advantages of the regenerative mechanism of axolotls and the current knowledge about the structures and functions of glycosaminoglycans for successful spinal cord regeneration and clinical applications (Bryant & Gardiner, 2018; Swarup, Hsiao, et al., 2013).

## 2 PATHOPHYSIOLOGY OF SPINAL CORD INJURIES

Currently, our knowledge of the pathophysiology of SCIs is based on animal models, mainly rodent studies. Therefore, SCIs acting through primary and secondary mechanisms will be discussed concisely below.

### 2.1 Primary injuries: disruption of the blood‐spinal cord barrier

Primary injuries result after physical trauma to the spinal cord, the most common being compression and contusion due to fractures or displacement of bone and discs in the spinal column (Tran et al., 2018). The physical trauma causes permeability to the blood‐spinal cord barrier (BSCB), which is responsible for keeping toxic products and other molecules excluded from the spinal cord. The BSCB, which is present at capillaries, mainly consists of endothelial cells and tight junctions, which controls transport of small and large molecules entering the CNS (Mautes, Weinzierl, Donovan, & Noble, 2000). The reason for the permeability could be explained by endothelin‐1, a vasoactive peptide with increased expression after injury, causing reduced blood flow in the spinal cord and subsequent cell damage (Mautes et al., 2000; Westmark, Noble, Fukuda, Aihara, & McKenzie, 1995). Although the impermeability of the BSCB is restored from 4–5 hr for large molecules to 4 days for small molecules, toxic molecules during these time periods pass through the BSCB, causing linked degeneration of axons and oligodendrocytes and the failure for neurons to conduct signals in animal models (Habgood et al., 2007; James et al., 2011). This could be the result of necrotic degeneration of neurons that extended beyond the impact site.

### 2.2 Secondary injuries: the glial scar dogma

The physical damage from primary injuries produce a longer‐lasting biological damage called secondary injury. Another complication with the permeability of the BSCB is that it also triggers an inflammatory response, which includes the release of alarmins and other molecules that respond to inflammation (Bianchi, 2007). The inflammatory response is highlighted by glial scarring, upregulation of inhibitory chondroitin sulfate proteoglycans, and astrocytic migration. This leads to the prevailing perspective that glial scars inhibit CNS axonal regrowth.

However, a recent study by Anderson et al. (2016) reported the opposite: that astrocytic scars promote axonal regeneration. They explored this by utilizing rodent models to identify axonal regrowth after preventing or removing scars and analyze chondroitin sulfate proteoglycans levels. The key recommendation made by the researchers is that astrocytes can be exploited to promote axonal regrowth (Anderson et al., 2016). This finding is against the perspective that glial scars cause inhibition of regeneration, but the glial scar is more than just astrocytes and the conclusions remain controversial in several aspects (Silver, 2016). Furthermore, chondroitin sulfate proteoglycans are predominant components of the perineuronal nets (PNNs) (Fawcett et al., 2019). Different sulfated chondroitin sulfate glycosaminoglycan structures in extracellular matrix either promote or inhibit the neural regeneration through multiple mechanisms (Swarup, Hsiao, et al., 2013).

## 3 GLYCOSAMINOGLYCANS IN AXONAL REGENERATION

Various mammalian neuronal cell types are surrounded by perineuronal nets (PNNs), which are chondroitin sulfate‐enriched cartilage‐like structures (Fawcett et al., 2019). A similar chondroitin sulfate‐enriched loose structure, the perinodal extracellular matrix, surrounds the axonal nodes of Ranvier. The negatively charged chondroitin sulfate in perinodal extracellular matrix also acts as an ion‐diffusion barrier that affects axonal conduction speed. Most importantly, injecting a bacterial enzyme that degrades chondroitin sulfate promotes axonal regeneration in a variety of animal models (Alilain, Horn, Hu, Dick, & Silver, 2011; Bradbury et al., 2002; Carter et al., 2008; Rosenzweig et al., 2019), which represents a feasible approach for human therapy in the near future. In addition to chondroitin sulfate, heparan sulfate glycosaminoglycans also play important roles in neuronal development and axonal regeneration (Inatani et al., 2003; Lander, Stipp, & Ivins, 1996; McCanney et al., 2019; Poulain & Yost, 2015).

### 3.1 Glycosaminoglycans and proteoglycans

Two major types of glycosaminoglycans are heparan sulfate and chondroitin sulfate in the form of proteoglycans where one glycosaminoglycan chain, such as in decorin, and up to 100 glycosaminoglycan chains, such as in aggrecan, are attached to the core protein of a proteoglycan (Zhang, 2010). Over 50 proteoglycan cDNAs have been cloned. Almost all cloned chondroitin sulfate and heparan sulfate proteoglycans, such as aggrecan, versican, brevican, neurocan, decorin, syndecans1‐4, glypicans 1–6, testican1‐2, perlecan, and agrin, have been found in the nervous system (Hartmann & Maurer, 2001). Because of the expression repertoire of the glycosaminoglycan assembly enzymes, each heparan sulfate and chondroitin chain has a sulfation pattern, chain length, and fine structure that is potentially unique to each cell. Around million copies of heparan sulfate and chondroitin sulfate are on the cell surface and the concentrations of heparan sulfate and chondroitin sulfate are at concentrations of ~mg/ml in the extracellular matrix (Lander & Selleck, 2000).

Glycosaminoglycans adopt an extended helical coil structure with a length ranging from 40 to 160 nm. Such abundance and size implies that glycosaminoglycans are a dominant feature of the cell surface glycocalyx (Tarbell & Cancel, 2016) and are an important feature of the extracellular matrix. Cell surface glycosaminoglycans turn over within 1/8 to 1/3 of a cell cycle (Zhang, 2010). This means their structures are able to rapidly change in response to a variety of environmental factors. Indeed, glycosaminoglycan structures are cell type‐specific (Sanderson, Turnbull, Gallagher, & Lander, 1994), which means different core proteins of proteoglycans produced by the same cells have the same glycosaminoglycan structures whereas the same proteoglycan core protein carries different glycosaminoglycan chains when produced by different types of cells.

**Figure 1:**
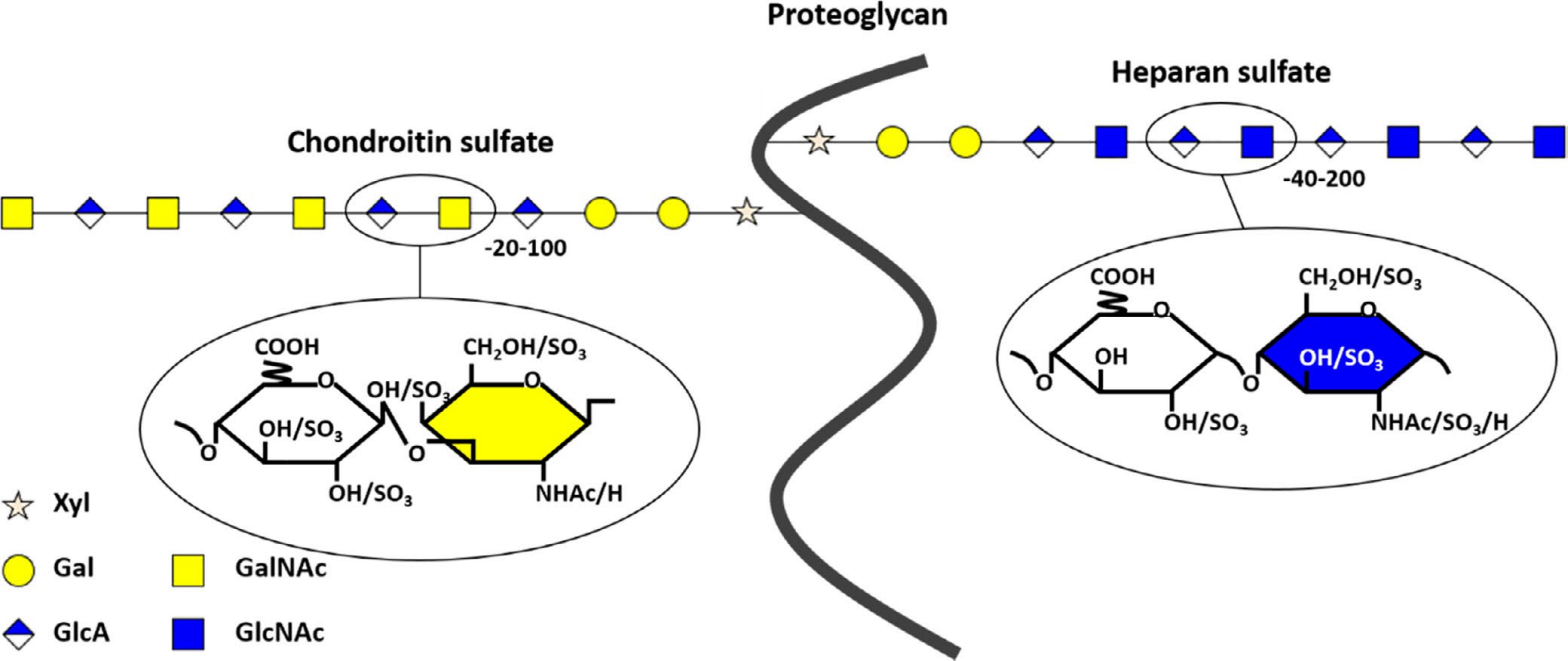
Heparan sulfate and chondroitin sulfate assembly on a proteoglycan core protein. Both heparan sulfate and chondroitin sulfate are attached to specific serine residues of proteoglycan core protein through the linkage tetrasaccharide GlcA (black)‐Gal (yellow)‐Gal (yellow)‐Xyl (pink). Biosynthesis starts with the transfer of xylose from UDP‐xylose to a serine residue of a core protein catalyzed by two xylosyltransferases. The linkage region is then synthesized by the sequential addition of two galactose residues (by galactosyltransferase I and II) and glucuronic acid (by glucuronosyltransferase I) from the corresponding UDP‐sugars. After completion of the linkage tetrasaccharides, the addition of GalNAc from UDP‐GalNAc by N‐acetylgalactosaminyl transferase I to the nonreducing terminal GlcA commits the intermediate to chondroitin sulfate biosysnthesis, which occurs subsequently through alternating addition of GlcA and GalNAc (green) by chondroitin synthase. If GlcNAc is added to the linkage tetrasaccharide instead by N‐acetylglucosaminyl transferase I, heparan sulfate synthesis occurs. Alternating GlcA and GlcNAc (red) residues are then added by heparan sulfate copolymerases (EXT‐1 and EXT‐2) from their corresponding UDP‐sugars. Overall, heparan sulfate and chondroitin sulfate are polymerized, epimerized, and sulfated by enzymes that are encoded by more than 40 genes (Zhang, 2010). Moreover, heparan sulfate proteoglycans always carry chondroitin sulfate. Chondroitin sulfate proteoglycans can contain small amount of heparan sulfate (Govindraj et al., 2002; Kresse et al., 2001). A universal symbol for the graphical representation of glycosaminoglycan structures in a proteoglycan was used in this figure, a modified version from the previous publication (Zhang, 2010)

Both heparan sulfate and chondroitin sulfate are assembled to specific Ser residues on the proteoglycan core protein through a tetrasaccharide linkage region, GlcA‐Gal‐Gal‐Xyl‐Ser (Figure 1). The synthesis of this region is initiated by the addition of a Xyl to Ser followed by the addition of two Gal residues and is completed by the addition of GlcA. The pathways of heparan sulfate and chondroitin sulfate synthesis depart after formation of the linkage tetrasaccharide. The addition of GlcNAc to the linkage tetrasaccharide commits to the assembly of heparan sulfate. Similarly, the addition of a GalNAc commits to the assembly of chondroitin sulfate. Chondroitin sulfate assembly on the linkage tetrasaccharide represents a default pathway. Heparan sulfate assembly requires special amino acid sequences proximal to the linkage tetrasaccharide (Esko & Zhang, 1996). The proportion of heparan sulfate and chondroitin sulfate carried on a heparan sulfate proteoglycan is cell type‐ (or tissue‐) dependent. For example, the only “absolute” heparan sulfate proteoglycan, glypican‐1, carries 90% heparan sulfate and 10% chondroitin sulfate when expressed in COS cells and 80% heparan sulfate and 20% chondroitin sulfate when expressed in CHO cells (Zhang, 2010). Low amounts of heparan sulfate have been detected on certain chondroitin sulfate proteoglycans such as in biglycan and aggrecan (Govindraj et al., 2002; Kresse et al., 2001). Thus, there is no absolute proteoglycan that carries only one type of glycosaminoglycan chains.

Despite that glycosaminoglycans have been used as clinical drugs for over 80 years (Hao, Xu, Yu, & Zhang, 2019) and also served as nutraceuticals (Zhang, 2019), the biological functions of glycosaminoglycans are largely overlooked until the geneticists discovered that the enzymes responsible for glycosaminoglycan biosynthesis and degradation are essential for animal development and are responsible for a series of hereditary human diseases (Bishop, Schuksz, & Esko, 2007). During the last 20 years, transgenic and knockout animal data provide compelling evidence that the structural diversity of glycosaminoglycans is a component of a sugar/sulfation code that imparts unique and specific biological functions during animal development (Zhang, 2010).

Chondroitin sulfate and heparan sulfate contain 20–400 repeating disaccharide units. Each disaccharide in heparan sulfate can be modified by N‐ and O‐sulfation (6‐O‐ and 3‐O‐sulfation of the glucosamine and 2‐O‐sulfation of the uronic acid) and epimerization of the glucuronic acid to iduronic acid with over 20 modification enzymes or enzyme isomers (Zhang, 2010). Each disaccharide in chondroitin sulfate can be modified by 4‐O‐ and 6‐O‐sulfation of the galactosamine and 2‐O‐ and 3‐O‐sulfation of the uronic acid and by epimerization of glucuronic acid to iduronic acid with over 16 modification enzymes or enzyme isomers (Zhang, 2010). In theory, five different types of disaccharide modifications can give rise to 32 possible disaccharide structures in either heparan sulfate or chondroitin sulfate. With 23 disaccharides found in chondroitin sulfate and 24 found in heparan sulfate (Esko & Selleck, 2002), a heparan sulfate or chondroitin sulfate hexasaccharide that binds to protein ligands could have several thousand possible sequences, which make them not only the most acidic but also the most information‐dense biopolymers in animal cell surface and in extracellular matrix (Zhang, 2010).

According to non‐, mono‐, di‐sulfated, the sulfation position, and the status of epimerizarion, chondroitin sulfate has been categorized as chondroitin sulfate A (4‐O‐sulfated), chondroitin sulfate B or dermatan sulfate (iduronic acid containing 4‐O‐sulfated chondroitin sulfate), chondroitin sulfate C (6‐O‐sulfated), chondroitin sulfate D (2, 6‐O‐disulfated), and chondroitin sulfate E (4, 6‐O‐disulfated) based on the major constituent of the repeating disaccharides. However, all chondroitin sulfates are hybrid structures that contain more than three types of disaccharides even from the same type of cells. Commercially available chondroitin sulfates A, B, C, D, and E have at least three types of disaccharides (Swarup, Hsiao, et al., 2013).

### 3.2 Chondroitin sulfate in axonal regeneration

Increased levels of chondroitin sulfate are a hallmark of all CNS injuries and have been shown to limit axonal plasticity, regeneration, remyelination, conduction, and to regulate immunity after injury (Silver & Miller, 2004). The pattern of sulfation of chondroitin sulfate in PNNs is different from that of the perinodal extracellular matrix (Deepa et al., 2006). Additionally, chondroitin sulfate creates a nonpermissive milieu for cell replacement activities by limiting cell migration, survival, and differentiation (Hayes & Melrose, 2018). However, some sulfation variants of chondroitin sulfate have been found in growth permissive regions of the CNS, and chondroitin sulfate can also stimulate neuron growth (Swarup, Hsiao, et al., 2013).

The sulfation pattern of chondroitin sulfate changes during brain development. Chondroitin sulfate in chicken embryonic brain is mostly 6‐sulfated (Kitagawa, Tsutsumi, Tone, & Sugahara, 1997). Immunohistochemistry revealed a progressive increase in chondroitin 4‐sulfate and decrease in chondroitin 6‐sulfate levels from 3 to 18 months (Maeda, 2010). In mice, 18% chondroitin sulfate is 6‐sulfated and 60% chondroitin sulfate is 4‐sulfated at birth. In contrast, 2.5% chondroitin sulfate is 6‐sulfated and 91.5% chondroitin sulfate is 4‐sulfated in adult mice (Carulli et al., 2010). Brain glycosaminoglycans extracted from PNNs show a large reduction in 6‐sulfated chondroitin sulfate from 12 to 18 months with increased 4‐sulfate/6‐sulfate ratio. PNN glycosaminoglycans are more inhibitory to axon growth than those from the perinodal extracellular matrix. The 18‐month PNN glycosaminoglycans are more inhibitory than 3‐month PNN glycosaminoglycans. Finally, in the aged rodent brain, 6‐sulfated chondroitin sulfate levels are diminished (Foscarin, Raha‐Chowdhury, Fawcett, & Kwok, 2017). These changes suggest that the 4‐sulfated and 6‐sulfated chondroitin sulfate levels might have very different properties during SCIs.

By using commercially available chondroitin sulfates A, B, C, D, and E where sulfation patterns in each type of chondroitin sulfate are not uniform (Swarup, Hsiao, et al., 2013), it was discovered that 4‐O‐sulfate‐enriched chondroitin sulfates are largely neurite attracting whereas 6‐O‐sulfate‐enriched chondroitin sulfates are largely neurite repelling. In addition, a combination of neurite attracting and repelling chondroitin sulfates in cell choice assay without any protein component is sufficient for directing neuronal outgrowth (Swarup, Hsiao, et al., 2013). These results indicate that different types of chondroitin sulfate serve different purposes in regulating neuronal growth, inhibition, and pathfinding.

### 3.3 Heparan sulfate in the development of the nervous system and in axonal regeneration

Using mouse and *Caenorhabditis elegans* models, it has been demonstrated that heparan sulfate plays multiple roles in the development of the nervous system involving generation of neurons from neural stem cells, migration of the generated neurons, extension of axons and dendrites, establishment of neuronal connectivity (Bulow & Hobert, 2004). *C. elegans* lacking heparan sulfate modifying enzymes, including glucuronyl C5‐epimerase, heparan 2‐O‐sulfotransferase, and 6‐O‐sulfotransferase exhibit distinct as well as overlapping axonal and cellular guidance defects in specific neuron classes, which are linked to two specific guidance pathways, the sax‐3/Robo and kal‐1/Anosmin‐1 systems in *C. elegans* (Bulow & Hobert, 2004). In contrast, generating stereotypical neurite branches in hermaphroditic‐specific neurons required heparan 3‐O‐sulfotransferases 3.1 and 3.2, as well as an extracellular cell adhesion molecule encoded by kal‐1, the homolog of Kallmann Syndrome associated gene 1/anosmin‐1. Interestingly, kal‐1‐dependent neurite branching in AIY neurons required catalytic activity of heparan 3‐O‐sulfotransferases 3.1 but not heparan 3‐O‐sulfotransferases 3.2. The context‐dependent requirement for 3‐O‐sulfotransferases 3.1 or 3.2 demonstrates that each enzyme generate specific heparan sulfate structure, which regulates kal‐1 to promote neurite branching, indicating heparan sulfate contains branching information for neurite.

Conditionally knocking out heparan sulfate polymerizing enzyme EXT1 in the embryonic mouse brain leads to patterning defects due to disrupted functions of multiple heparan sulfate‐binding morphogens, indicating that heparan sulfate is a patterning molecule required for midline axon guidance in the mouse model (Inatani et al., 2003; Yamaguchi, Inatani, Matsumoto, Ogawa, & Irie, 2010).

In cell‐based model systems, heparan sulfate supports neurite outgrowth through interacting with growth‐enhancing growth factors and extracellular matrix proteins, such as heparin‐binding EGF, NCAM, laminin, and several midkines (Zhou & Besner, 2010). In vivo studies showed that the expression of heparan sulfate proteoglycans, such as cerebroglycan (Ivins, Litwack, Kumbasar, Stipp, & Lander, 1997), syndecan (Hsueh & Sheng, 1999), and glypicans (Saunders, Paine‐Saunders, & Lander, 1997), is closely associated with neurite outgrowth.

Most of research on the role of glycosaminoglycans on axonal regeneration has been focused on chondroitin sulfate. However, Barnett's laboratory has developed myelinating cultures (Sorensen, Moffat, Thomson, & Barnett, 2008) to test the role of heparan sulfate mimetics in remyelination and/or neurite outgrowth to study such aspects of SCIs (McCanney et al., 2019). Their results showed that N‐sulfated heparan sulfate mimetics promote myelination whereas O‐sulfated heparan sulfate mimetics do not affect myelination but promote neurite outgrowth (McCanney et al., 2019). Again, these findings demonstrated that different sulfation patterns in heparan sulfate play different roles for axonal regeneration.

Synapses are fundamental units of communication in the brain. Zhang et al reported recently that neurexin‐1 is a heparan sulfate proteoglycan and mice lacking heparan sulfate on the neurexin‐1 core protein have reduced survival rates and functional deficits at the central synapses, which demonstrate that heparan sulfate organizes neuronal synapses through neurexin partnerships (Zhang et al., 2018).

Chondroitin sulfate inhibits axonal growth while heparan sulfate promotes it. Griffith et al showed that heparan sulfate, chondroitin sulfate D and E, but not chondroitin sulfate A, bind to RPTPσ, NgR1, NgR2, and NgR3 with high affinity based on both the glycosamaminoglycan‐dock computational method and direct binding assays (Griffith et al., 2017). They further demonstrated that the predicted structure contains multiple solvent‐exposed sulfate groups for heparin, whereas the predicted chondroitin sulfate E structure has all sulfate groups oriented toward the glycosaminoglycan binding site of RPTPσ. These differences could allow the heparin–RPTPσ complex to engage an additional RPTPσ through these solvent‐exposed sulfate groups. The distinct RPTPσ binding patterns for heparan sulfate and chondroitin sulfate might explain their different effects on axonal regeneration. A recent work by Sakamoto et al. also showed that PTPRσ interacts with oligomers of both chondroitin sulfate E and heparan sulfate. Chondroitin sulfate E activates PTPRσ, which dephosphorylates cortactin and disrupts autophagy flux at the autophagosome‐lysosome fusion step. Such disruption is required and sufficient for the dystrophic endball formation and inhibition of axonal regeneration (Sakamoto et al., 2019).

In summary, both chondroitin sulfate and heparan sulfate glycosaminoglycans have enormous structural diversity due to their nontemplate driven biosynthesis (Esko & Selleck, 2002; Xu & Esko, 2014; Zhang, 2010). Genetic studies have demonstrated that loss of specific modification enzymes during chondroitin or heparan sulfate glycosaminoglycan biosynthesis can lead to catastrophic neuronal defects (MaedaJul , Ishii, Nishimura, & Kamimura, 2011; Silver & Silver, 2014; Townley & Bulow, 2018). It is known that growing axons are guided toward their targets by the combined actions of attractants and repellents, where specific glycosaminoglycan structures serve such functions. Both genetic and biochemical studies provide compelling evidence that glycosaminoglycans are long‐sought patterning molecules responsible for axonal regeneration. Thus, translating such knowledge into medical practice for patients suffering SCIs will be the next challenge.

## 4 AXOLOTLS AS SPINAL CORD REGENERATION MODELS

Almost all salamanders, in addition to axolotls, can regenerate their spinal cord after injury, including several newt models and fish species. The main reason axolotls are used in laboratory research is that they can be bred in captivity, which was not the case for any other salamander until recently. This makes axolotls the highest throughput model and amenable to genetic and other studies.

**Figure 2:**
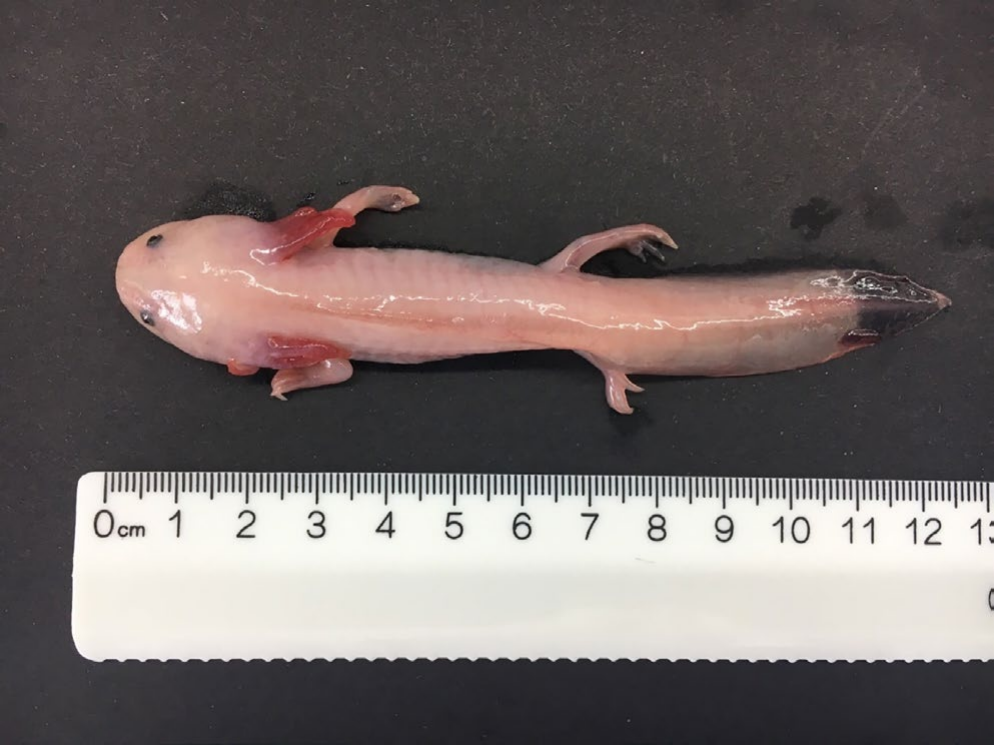
A leucitic axolotl, resulting from a recessive mutation

The lack of effective treatment for spinal cord injuries is the main reason for using axolotls as a potential spinal cord regeneration model. Axolotls (scientific name *Ambystoma mexicanum*), native to Xochimilco and Chalco lakes in Mexico City, are unique that they do not undergo metamorphosis and fully regenerate body parts without scarring (Lab Anim, 2012; Menger, Vogt, Kuhbier, & Reimers, 2010). They also have different colors, including the darker wild‐type and a leucitic color resulting from a recessive mutation. For research purposes, leucitic axolotls (Figure 2) are preferred since they do not interfere with staining and imaging (Farkas & Monaghan, 2015). Despite being critically endangered in the wild, axolotls are easy to breed. Furthermore, despite the extraordinary regeneration capacity, conserved signaling pathways regulate regeneration, thereby meaning that findings involving axolotls should be applicable to humans (McCusker & Gardiner, 2011). In this section, we will discuss the current use of axolotls in the laboratory and specifically in terms of spinal cord regeneration.

### 4.1 Axolotls in the laboratory

Laboratory axolotls of today are mostly descendants of those brought to Paris in 1863 (Lab Anim, 2012). Unlike almost all vertebrates, axolotls can regrow complete body structures after amputation, including components such as skin, nerves, and muscle (Denis, Levesque, Tran, Camarda, & Roy, 2013). Limb regeneration research identified progenitor cells that help the cells remain their original identity during regeneration (Kragl et al., 2009; Lab Anim, 2012). Another potential area of research is wound healing, due to their ability to have scar‐free healing even as an adult and is potentially an important model for plastic surgery (Menger et al., 2010). However, whether these results apply to humans remain unsolved, not only for limb regeneration, but also for spinal cord regeneration.

However, there are several limitations involving the use of axolotls. Axolotls remain in neoteny (i.e., underdeveloped larval stage) for the entirety of their life, meaning that they do not develop adaptive immunity (Menger et al., 2010; Mescher & Neff, 2006). Although this means that they remain aquatic animals, their ability to regenerate remains despite that the experimentally induced metamorphosis in axolotls reduces regenerative rate and fidelity (Monaghan et al., 2014). The human immune system plays a major role in wound healing and therefore not translatable to axolotl physiology (Menger et al., 2010). However, a counterargument could be made that genes critical to development and regeneration are strongly conserved, meaning that that genes that cause regeneration in axolotls can be present in humans, albeit inhibited (Denis et al., 2013). Therefore, a future research direction, regardless of field, could be identifying and activating the regenerating process found in axolotls.

### 4.2 Current spinal cord regeneration leads involving axolotls

As mentioned earlier, SCIs in mammals result in a failure to regain sensory and motor function due to glial scarring and other inhibitory mechanisms. This results in the use of axolotls and other salamanders for spinal cord regeneration research, since they can regenerate the spinal cord and retaining the integrity of structure and function. Two axolotl models used commonly in current literature are transection and amputation models, although transection studies are slightly more pertinent to mammalian models (Chernoff, Sato, Salfity, Sarria, & Belecky‐Adams, 2018). A recent review by Tazaki et al. (2017) emphasized that in both adult mammals and salamanders, there are differences in non‐neural and glial cell populations, which emphasizes the potential contributions of different cell populations in the spinal cord regeneration.

Gardiner's laboratory showed for the first time that heparan sulfate in the extracellular matrix has positional information required to induce formation of new limb pattern during regeneration in axolotls (Phan et al., 2015). In the accessory limb model of axolotls, they demonstrated that cells in ectopic blastemas respond to signals associated with the cell‐free extracellular matrix and formed ectopic limb structures. The ability of cell‐free axolotl limb extracellular matrix to control pattern formation is position‐specific in that posterior, not anterior, extracellular matrix induces pattern formation in anterior blastemas. In contrast, anterior extracellular matrix inhibits blastema formation (Phan et al., 2015). The observed difference is dependent on different heparan sulfate structures that are associated with differential expression of heparan sulfate sulfotransferases. Moreover, an artificial extracellular matrix containing only heparan sulfate is sufficient to induce de novo limb pattern in axolotl limb regeneration. Furthermore, extracellular matrix from mouse limbs is capable of inducing limb pattern in axolotl blastemas in a position‐specific, developmental‐stage‐specific, and heparan sulfate‐dependent manner. Furthermore, Sahu et al demonstrated that knockdown of chondroitin‐4‐sulfotransferase‐1, but not of dermatan‐4‐sulfotransferase‐1, accelerates regeneration of zebrafish after spinal cord injury, indicating that chondroitin sulfate and dermatan sulfate structures play different roles in axonal regeneration (Sahu, Li, Loers, & Schachner, 2019). When using immunohistochemistry to examine the expression of two chondroitin sulfates with different sulfation variants at the lesion site in the spinal cord of goldfish, Takeda et al showed that chondroitin sulfate is co‐localized with the regenerating axons (Takeda, Okada, & Funakoshi, 2017). Thus, chondroitin sulfate contributes to spinal cord regeneration after injury in zebrafish and goldfish as well.

### 4.3 How axolotls can influence future regeneration research

Farkas et al reported that neuregulin‐1 signaling is essential for nerve‐dependent axolotl limb regeneration (Farkas, Freitas, Bryant, Whited, & Monaghan, 2016). Interestingly, Pankonin et al showed that specific heparan sulfate structures potentiate neuregulin‐1 signaling (Pankonin, Gallagher, & Loeb, 2005). While retinoic acid receptor regulation of epimorphic and homeostatic regeneration is present in the axolotl (Nguyen et al., 2017), retinoic acid alone or a combination of retinoic acid and cAMP plus theophylline trigger F9 cells to differentiate into parietal endoderm, which induces a ninefold increase in total heparan sulfate biosynthesis and a 170‐fold increase in anticoagulantly active heparan sulfate structure biosynthesis (Zhang et al., 1998). It would be remarkable to test whether the retinoic acid signaling also induces augmented heparan sulfate biosynthesis in axolotls and how heparan sulfate subsequently impacts spinal cord regeneration. However, only a few laboratories worldwide perform glycosaminoglycan structural analysis worldwide. Studies from these laboratories will be needed to understand if specific glycosaminoglycan structures regulate spinal cord regeneration through different cellular signaling pathways.

The studies using axolotls as model systems indicate specific heparan sulfate structures, growth factors (especially FGFs and BMPs), and regeneration‐competent cells are the key elements for regeneration (Bryant & Gardiner, 2018; Makanae, Mitogawa, & Satoh, 2016; Silver & Silver, 2014). Most importantly, the extracellular component heparan sulfate can induce de novo limb pattern formation during regeneration and heparan sulfate in mediating positional information is conserved in mammals (Phan et al., 2015). A recent study also showed that chondroitin sulfate is the major retinal glycosaminoglycan followed by heparan sulfate in both native and decellularized axolotl and porcine retina (Kim et al., 2019). Higher levels of 4‐O‐ and 6‐ O‐sulfation are observed in axolotl retina compared with that in porcine retina. Different heparan sulfate sulfation patterns in the retina are also evident between axolotl and porcine. The overall results suggest the unique glycosaminoglycan composition and structures of the axolotl retina might set foundation for axolotl retina regeneration.

## 5 CONCLUSION

The explosive growth of information from the studies of genetics and genomics in different animal models have revealed that the key signaling networks and key molecules that control both development and regeneration are highly conserved. Among them, the indirect gene products, heparan sulfate and chondroitin sulfate glycosaminoglycans, are among the key players required for animal development and regeneration as patterning molecules. Thus, the diversity of biological processes among animals is not a consequence of the differences in signaling networks, but differences in regulation of conserved signaling networks in time and space through glycosaminoglycans, the complex biomolecules assembled and modified by hundreds of enzymes, and environmental factors. However, in contrast to growth factors and morphogens, glycosaminoglycans are much more abundant and structurally stable, which allow them to be isolated in sufficient amounts to engineer glycosaminoglycan‐based matrices that might make spinal cord regeneration possible. Indeed, patients suffering SCIs are in urgent need of effective regenerative therapies. By combining the insights provided by developmental biologists, lessons learned from axolotls, specific glycosaminoglycan structural information provided by glycobiologists, and technologies developed by biomaterial engineers, spinal cord regeneration in humans should be possible in the near future.

## CONFLICT OF INTEREST

The authors declare no conflict of interest.

## Data Availability

*Brain and Behavior* expects that data supporting the results in the paper will be archived in an appropriate public repository. Authors are required to provide a data availability statement to describe the availability or the absence of shared data. When data have been shared, authors are required to include in their data availability statement a link to the repository they have used, and to cite the data they have shared. Whenever possible the scripts and other artefacts used to generate the analyses presented in the paper should also be publicly archived. If sharing data compromises ethical standards or legal requirements then authors are not expected to share it. Data available on request from the authors.
